# Trophic consequences of introduced species: Comparative impacts of increased interspecific versus intraspecific competitive interactions

**DOI:** 10.1111/1365-2435.12978

**Published:** 2017-09-21

**Authors:** J. Robert Britton, Ana Ruiz‐Navarro, Hugo Verreycken, Fatima Amat‐Trigo

**Affiliations:** ^1^ Faculty of Science and Technology Bournemouth University Poole UK; ^2^ Research Institute for Nature and Forest (INBO) Brussels Belgium; ^3^ Departmento de Zoología y Antropología Física Universidad de Murcia Murcia Spain

**Keywords:** biological invasions, global change, isotopic niche, niche divergence

## Abstract

1. Invasive species can cause substantial ecological impacts on native biodiversity. While ecological theory attempts to explain the processes involved in the trophic integration of invaders into native food webs and their competitive impacts on resident species, results are equivocal. In addition, quantifying the relative strength of impacts from non‐native species (interspecific competition) versus the release of native conspecifics (intraspecific competition) is important but rarely completed.
2. Two model non‐native fishes, the globally invasive *Cyprinus carpio* and *Carassius auratus*, and the model native fish *Tinca tinca*, were used in a pond experiment to test how increased intra‐ and interspecific competition influenced trophic niches and somatic growth rates. This was complemented by samples collected from three natural fish communities where the model fishes were present. The isotopic niche, calculated using stable isotope data, represented the trophic niche.
3. The pond experiment used additive and substitutive treatments to quantify the trophic niche variation that resulted from intra‐ and interspecific competitive interactions. Although the trophic niche sizes of the model species were not significantly altered by any competitive treatment, they all resulted in patterns of interspecific niche divergence. Increased interspecific competition caused the trophic niche of *T. tinca* to shift to a significantly higher trophic position, whereas intraspecific competition caused its position to shift towards elevated δ^13^C. These patterns were independent of impacts on fish growth rates, which were only significantly altered when interspecific competition was elevated.
4. In the natural fish communities, patterns of trophic niche partitioning between the model fishes was evident, with no niche sharing. Comparison of these results with those of the experiment revealed the most similar results between the two approaches were for the niche partitioning between sympatric *T. tinca* and *C. carpio*.
5. These results indicate that trophic niche divergence facilitates the integration of introduced species into food webs, but there are differences in how this manifests between introductions that increase inter‐ and intraspecific competition. In entirety, these results suggest that the initial ecological response to an introduction appears to be a trophic re‐organisation of the food web that minimises the trophic interactions between competing species.

A plain language summary is available for this article.

## 1 INTRODUCTION

Biological invasions are a substantial driver of global environmental change that have major implications for native biodiversity (Simberloff et al., 2013). When an introduced species establishes a population, then ecological impacts on native species can be incurred through competitive interactions (Gozlan, Britton, Cowx, & Copp, 2010). These impacts can be particularly strong where the invader and native species are closely related (Li et al., 2015; Ricciardi & Atkinson, 2004) or functionally similar (Dick et al., 2016, 2017), as the species are more likely to be foraging on the same food resources (Buoro, Olden, & Cucherousset, 2016).

A number of niche‐based hypotheses have been suggested to explain the processes that facilitate the development of successful invasions and enable native species to coexist trophically with the invader (Ricciardi, Hoopes, Marchetti, & Lockwood, 2013). For example, the utilisation of unexploited resources by an invader would avoid competitive interactions with native species and so facilitate their integration into the food web (Juncos, Milano, Macchi, & Vigliano, 2015; Mason, Irz, Lanoiselée, Mouillot, & Argillier, 2008; Okabe & Agetsuma, 2007). In situations where resources are fully exploited then niche theory predicts that competitive interactions between invasive and native species will result in both their niches being smaller than in allopatry (Bolnick et al., 2010; Jackson, Grey, et al., 2016; Tran et al., 2015a, 2015b). Conversely, increased interspecific competition might result in species increasing their niche breadths to maintain their energy requirements (Svanbäck & Bolnick, 2007). Moreover, where the non‐native species is a superior competitor, they can compete for the same resources as used by native species and result in the competitive exclusion of that native species (Tran et al., 2015a, 2015b). This can cause niche shift or replacement in the native species, leading to reduced food intake, slower growth rates and/or reduced population density (Bøhn, Amundsen, & Sparrow, 2008).

Ecological impacts resulting from increased competitive interactions due to introductions are, however, not limited to non‐native species. Impacts can also develop when the population of a native species increases in abundance. While this can occur naturally through strong recruitment, it also occurs when conspecifics are released to increase population size (‘stocking’) (Bašić & Britton, 2016). In fishes of the Salmonidae family, ecological impacts from stocking with conspecifics are often stronger than those resulting from non‐native fishes (Buoro et al., 2016). This is due to the ‘pre‐adaptation hypothesis’ where the released fishes have almost identical functional traits as resident species and so have a similar ability to acquire resources (Ricciardi & Mottiar, 2006). The subsequent increase in intraspecific competition then potentially results in similar ecological consequences to those resulting from interspecific competition (Carey, Sanderson, Barnas, & Olden, 2012), and can be tested using the similar hypotheses (Bøhn et al., 2008; Ricciardi et al., 2013; Svanbäck & Bolnick, 2007). However, differences in the processes by which ecological impacts result from intra‐ vs. interspecific competitive interactions remain poorly understood for many taxa.

The aim of this study was to therefore test the trophic consequences for populations of a model native species from increased intra‐ and interspecific competitive interactions that result from introductions. The model species were freshwater fishes, as they are adaptable and tractable experimental animals that provide excellent model systems for competitive studies (Ward, Webster, & Hart, 2006). For example, their indeterminate nature of growth enables correlation with competitive success (Ward et al., 2006). The model native species was tench *Tinca tinca*, a fish of the Cyprinidae family with a native range across most of Eurasia (Fishbase, 2017). Its rationale for use as a model was that its omnivory and broad environmental tolerances potentially provide highly plastic responses to increased competition (Guo, Sheath, Amat‐Trigo, & Britton, 2016). As the drivers of invasion success of non‐native fishes include their functional similarities to many native fishes, then the model species used to increase interspecific competition were the global cyprinid invaders carp *Cyprinus carpio* and goldfish *Carassius auratus*. Both fishes are primarily benthic foragers that exploit a wide range of trophic resources and so they are trophic analogues of *T. tinca* (Guo et al., 2016; Weber & Brown, 2011). All of the model fishes are exploitative competitors and thus were assumed to overlap in their diet in situations where food resources are limited.

Understanding the trophic interactions of invasive and native fishes is enhanced when experimental approaches are coupled with studies of invaded natural communities as, in combination, they enable ecological patterns and processes to be understood over a range of temporal and spatial scales (Tran et al., 2015a, 2015b). Consequently, using stable isotope analysis (SIA; δ^13^C, δ^15^N) to determine trophic relationships, the model species were initially used in a pond experiment based on additive and substitutive treatments. This experiment tested the following predictions: (1) competitive interactions between the model fishes significantly alters the size of their trophic niches and reduces their somatic growth rates; and (2) impacts from inter‐ and intraspecific competition are similar on the size and position of the trophic niche of the native species. Then, samples of the model fishes collected from invaded natural communities tested the prediction that: (3) the trophic relationships of the model species are similar in the experimental simulations and their populations in the wild.

## 2 MATERIALS AND METHODS

### 2.1 Experimental design

The experimental design (hereafter referred to as the ‘experiment’) used 10 additive and substitutive treatments across a combination of allopatric and sympatric contexts, with each treatment replicated three times (Table 1). Three control treatments used each species in allopatry (‘Allopatry’; *N* = 8; Table 1). Three substitutive treatments then paired the native and non‐native species in their different sympatric combinations (‘Sympatry’; *n* = 4 + 4, *N* = 8; Table 1). It was these three treatments whose isotopic niches of the sympatric fishes were compared to those from the invaded natural communities (Tables 1 and 2). Three additive treatments then used all three species in sympatry across three different abundances (‘Interspecific competition’; *n* = 4 + 4 + 4, 8 + 8 + 8, 12 + 12 + 12; *N* = 12, 24, 36; Table 1). Finally, a single treatment used the native species in higher abundance (‘Intraspecific competition’; *N* = 12). All the fish used in the treatments were juveniles and had been hatchery reared. As their starting lengths were 45–60 mm and starting weights <10 g, the predicted stable isotope half‐life for their dorsal muscle was 36 and 38 days for δ^13^C and δ^15^N respectively (Thomas & Crowther, 2015).

**Table 1:** Structure of the treatments used in the experiment, showing the number of fish per species per treatment (*n*), and the total number of fish per treatment (*N*)

Treatment	*Tinca tinca* (*n*)	*Cyprinus carpio* (*n*)	*Carassius auratus* (*n*)	*N*
Allopatry (*T. tinca*)	8	0	0	8
Allopatry (*C. carpio*)	0	8	0	8
Allopatry (*C. auratus*)	0	0	8	8
Sympatry (*T. tinca* + *C. carpio*)	4	4	0	8
Sympatry (*T. tinca* + *C. auratus*)	4	0	4	8
Sympatry (*C. carpio* + *C. auratus*)	0	4	4	8
Intraspecific competition	12	0	0	12
Interspecific competition (4)	4	4	4	12
Interspecific competition (8)	8	8	8	24
Interspecific competition (12)	12	12	12	36

**Table 2:** Details of the invaded communities (Sites 1–3), including their locations, sizes and information on the fish populations present

Site	Country	Location	Size (m^−2^)	Comparator model species (mean length ± 95% CI, mm)	Other fishes present	Reference
1	Wales	N: 51°41′10.0″ W: 4°12′06.00″	3000	*Tinca tinca* (96 ± 20) *Carassius auratus* (60 ± 4)	*Scardinius erythrophthalmus; Pseudorasbora parva*	Tran et al. (2015a, 2015b)
2	Belgium	N: 51°2′7.76″ E: 4°10′40.84″	1900	*Cyprinus carpio* (70 ± 6) *Carassius* spp. (86 ± 10)	*S. erythrophthalmus; Blicca bjoerkna; Rutilus rutilus; Leucaspius delineates; Rhodeus amarus*	Tran et al. (2015a, 2015b)
3	England	N: 51°12″ W: 0°34″^a^	3000	*T. tinca* (174 ± 20) *C. carpio* (218 ± 72)	*P. parva*	Jackson and Britton (2013)

a Approximate location as exact location unable to be provided for business confidentially reasons relating to *P. parva* invasion and subsequent eradication.

The experiment was completed using the treatments within enclosures that sat within a larger, man‐made pond (30 × 30 m; 1 m consistent depth) that was located in southern England. Following Bašić and Britton (2016), the enclosures comprised of an aluminium frame (length 1.66 m; width: 1.05 m; height: 1.2 m) within a net (mesh: 7 mm^2^) that prevented fish in‐ and egress, but allowed movements of invertebrates. The enclosures were placed randomly across the pond, with at least 0.5 m between them; they were sufficiently heavy that they remained in situ throughout the experimental period without movement and they sat on the substrate, with macrophytes able to grow within each of them (primarily *Elodea* spp.). Bird predation was prevented via netting over the enclosures (15‐mm mesh). The experiment ran for 150 days from April 2016. This duration enabled fish dorsal muscle to undergo approximately 4 half‐lives and so by its conclusion, the δ^13^C and δ^15^N data of the experimental fish would represent their diet in the ponds (Thomas & Crowther, 2015). All fish were weighed (nearest 0.1 g) prior to release into the enclosures. Temperature loggers (TinyTag TGP‐4017) in the larger pond revealed the mean water temperature was 18.1 ± 0.6°C during the experiment. On day 150, all the fish were recovered from the enclosures, euthanised (anaesthetic overdose, MS‐222) and taken to the laboratory. For stable isotope analysis, macroinvertebrate and macrophyte samples were taken from the larger pond, sorted into samples (one sample = 3–9 individuals per species), with triplicate samples taken.

### 2.2 Invaded wild fish communities

Three wild pond fish communities (hereafter referred to as the ‘invaded communities’) were used, with each having two of the model species present within a mixed community of other fishes (Table 2). At each site, the model fishes had been present for at least 5 years prior to sampling. Although data from each of these fish communities have been reported previously, the data used in this paper have not previously been compared (Table 2). Note that replicates of each combination of species were not used due to inherent logistical difficulties of locating sites were each species was present and sufficiently abundant to provide adequate sample sizes. Also, at Site 2, while *C. carpio* was present in sympatry with a *Carassius* species, this was identified in the field as *Carassius gibelio*. However, Busst and Britton (2017) indicated that *Carassius* species generally have high trophic similarity due to their similar functional traits (Busst & Britton, 2017) and thus *C. gibelio* was used as a surrogate of *C. auratus* at Site 2. Site 1 and 2 was sampled in spring 2013, whereas Site 3 was sampled in March 2008. At each site, fish sampling incorporated electric fishing, seine nets, fish traps and fyke nets. Following their capture, the fish were euthanised and returned to the laboratory for processing. The sample size for stable isotope analysis was a minimum of 10 individuals per species, with individuals randomly selected across the length range sampled (Table 2). As these were fish sampled from the wild, then this random selection resulted in a wider length range of fish being used than was the case in the experiment (Table 2).

### 2.3 Stable isotope analysis

In the laboratory, fish from the experiment and the invaded communities were measured and weighed, and a dorsal muscle sample taken for stable isotope analysis (SIA). SIA sample sizes were 10 fish per species for Sites 1 and 2, and 15 per species for Site 3. Along with the macroinvertebrate samples, all samples were dried at 60°C to constant mass before stable isotope analysis (SIA) (δ^13^C, δ^15^N) at the Cornell University Stable Isotope Laboratory, New York, USA, where they were ground to powder and weighed precisely to *c*. 1 000 μg in tin capsules and analysed on a Thermo Delta V isotope ratio mass spectrometer (Thermo Scientific, USA) interfaced to a NC2500 elemental analyser (CE Elantach Inc., USA). Analytical precision associated with the δ^15^N and δ^13^C sample runs was estimated at 0.42‰ and 0.15‰ respectively. Data outputs were in the format of delta (δ) isotope ratios expressed per mille. There was no lipid correction applied to the data as C:N ratios indicated very low lipid content (Post et al., 2007).

### 2.4 Data analysis

The SIA data from the experiment and invaded communities were used to calculate the trophic niche size of each fish species using the isotopic niche (Jackson, Inger, Parnell, & Bearhop, 2011). While closely related to the trophic niche, the isotopic niche is also influenced by factors including growth rate and metabolism, and thus represents a close approximation of the trophic niche (Jackson et al., 2011). It was calculated using standard ellipse areas (SEA) in SIBER (Jackson et al., 2012), a bivariate measure of the distribution of individuals in isotopic space; as each ellipse encloses ≈40% of data, they reveal the population's typical resource use (Jackson et al., 2011). The generally small sample sizes used in both study components (i.e. <30) meant a Bayesian estimate of SEA (SEA_B_) was used to test differences in niche sizes between species, calculated using a Markov chain Monte Carlo simulation (10^4^ iterations per group) (Jackson et al., 2012). Where 95% confidence intervals of SEA_B_ overlapped between comparator species, the isotopic niches were interpreted as not being significantly different in size. The stable isotope data were then used to calculate isotopic niche overlap (%) between the species in each treatment and across treatments using SEA_c_ calculated in SIBER, where subscript ‘c’ indicates a small sample size correction was used (Jackson et al., 2012). Use of SEA_c_ was only to get a representation of the extent of niche overlap between species, as it is more strongly affected by small sample sizes <30 than SEA_B_ (Jackson et al., 2012; Syväranta, Lensu, Marjomäki, Oksanen, & Jones, 2013).

For the invaded communities, SEA_B_ and SEA_c_ was calculated for each model species and compared between the species within each site, but not between sites due to the multiple context dependencies that can influence niche sizes between wild populations (Tran et al., 2015a, 2015b). For the experiment, as the treatments were completed within the same larger pond, all the fish had the same isotopic baseline and thus their SI data and niche data were able to be compared between species and treatments without any correction. Data per species were combined from replicates for each treatment to provide representative sample sizes sufficient for analyses of SEA_B_ and SEA_c_. A minimum of four randomly chosen individuals was used from each replicate to provide both a balanced dataset across the experiment and a minimum sample size per treatment of 12 fish per species (Table 1; Appendix S1: Figures S1–S3).

In the experiment, to then test differences in the SI data between species and treatments, δ^15^N was converted to trophic position (TP) from TP_i_ = [(δ^15^N_i_ − δ^15^N_base_)/3.4] + 2, where TP_i_ = trophic position of the individual fish, δ^15^N_i_ = fish isotopic ratio, δ^15^N_base_ = macroinvertebrate isotopic ratio, 3.4 = discrimination between trophic levels and 2 = trophic position of baseline macroinvertebrates (Jackson & Britton, 2014). TP and δ^13^C data were used in linear mixed effects models (LMEM) to test differences between treatments per species, with enclosure used as a random effect on the intercept to avoid inflating the degrees of freedom that would occur if individual fish were used as true replicates (Tran et al., 2015a, 2015b). The starting mass of fish in the enclosure was also initially used as a covariate, but was removed from all final models due to its effects not being significant (*p* > .05 in all cases). For each model, differences between species and treatment were assessed using estimated marginal means and linearly independent pairwise comparisons with Bonferroni adjustment for multiple comparisons.

To determine fish growth rates in the experiment, the mean specific growth rate (SGR) per model species and replicate was determined from: [(lnW_*t*+1_) − (lnW_*t*_)⁄n]/_*t*_, where W_*t*_ = total starting weight, W_*t*+1_ = total end weight, *n* = the number of fish used to determine W, and *t* = the duration of the experiment (days). A generalised linear model (GLM) tested the differences in SGR between treatments for each species. In each GLM, SGR was the dependent variable and treatment was the independent variable; total starting mass of fish per replicate initially used as a covariate and was retained in the final model when its effect on SGR was significant.

## 3 RESULTS

### 3.1 Trophic impacts of interspecific competition from non‐native species

**Figure 1:**
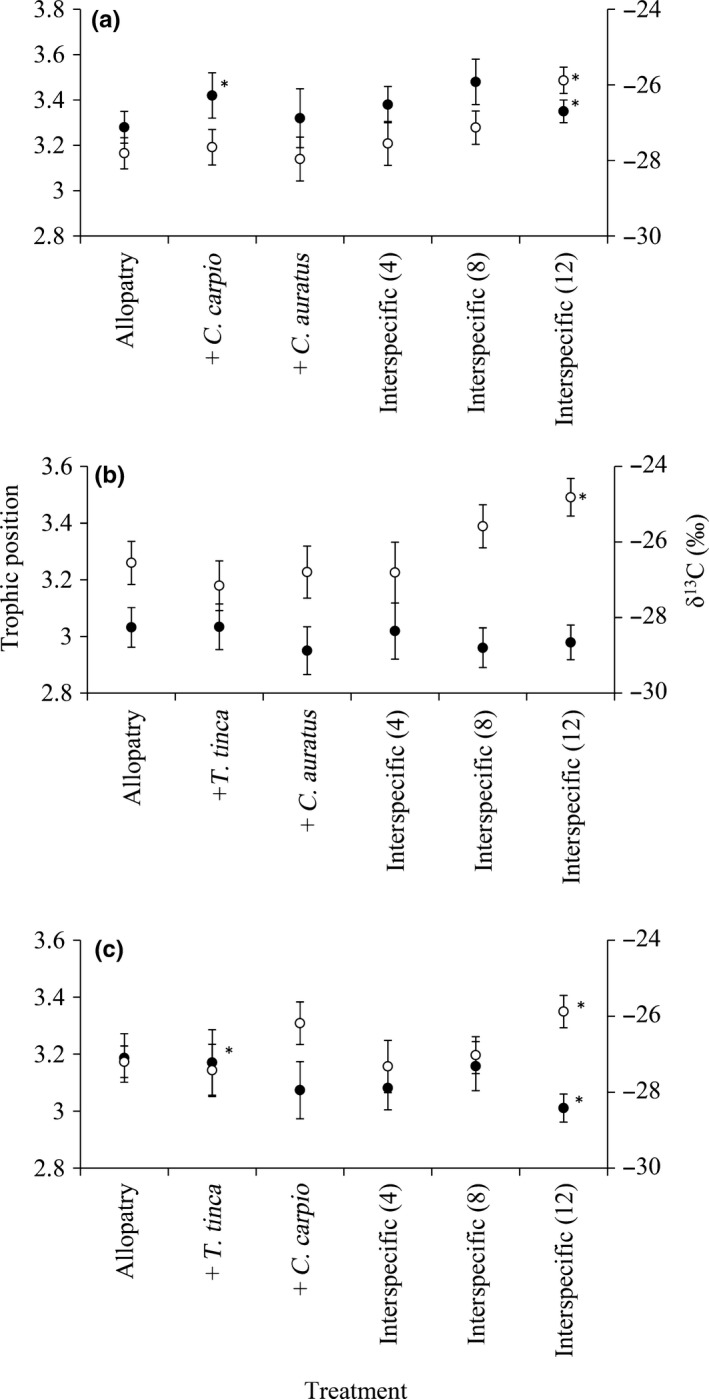
Mean δ^13^C (clear circle) and trophic position (filled circle) per experimental treatment for *Tinca tinca* (a), *Cyprinus carpio* (b) and *Carassius auratus* (c). *Difference between Allopatry and the treatment is significant at *p* < .05. Error bars represent 95% confidence limits

Across all of the experimental treatments, the isotopic niche sizes (as SEA_B_) of each species varied, but their 95% confidence intervals always overlapped between allopatry and sympatry, indicating no significant differences in isotopic niche size caused by the competition scenarios (Table 3). The LMEMs testing differences in TP and δ^13^C between treatments for each species were significant (*p* < .01), except TP in *C. carpio* (*p* = .47) (Tables S1–S3). Pairwise comparisons revealed the significant shifts TP and δ^13^C were mainly between the allopatric treatments and the interspecific competition (8) and (12) treatments (*p* < .05; Figure 1; Tables S1–S3). In the interspecific competition treatments, the pattern for *T. tinca* was a shift to significantly higher TP and higher δ^13^C, for *C. carpio*, the only shift was to significantly higher δ^13^C, and for *C. auratus*, the significant shifts were to lower TP and higher δ^13^C (Figure 1). Where shifts were to elevated values of δ^13^C, the fish were moving towards using macrophyte as an energy source (mean δ^13^C: −24.37‰ ± 0.88‰), away from macroinvertebrate prey resources (chironomid larvae, Corixidae, Odonata and Ephemeroptera: mean δ^13^C: −30.57‰ ± 1.28‰).

**Table 3:** Isotopic niche size (as lower and upper 95% confidence intervals of SEA_B_) of each species per treatment in the experiment

Treatment	Species		
	*Tinca tinca*	*Cyprinus carpio*	*Carassius auratus*
Allopatry	0.63–1.48	0.42–1.19	0.74–1.84
Sympatry (*T. tinca* + *C. carpio*)	0.50–1.40	1.08–3.88	—
Sympatry (*T. tinca* + *C. auratus*)	0.29–1.06	—	0.32–1.07
Sympatry (*C. carpio* + *C. auratus*)	—	0.73–2.96	0.69–2.33
Interspecific competition (4)	0.41–1.37	0.39–1.33	0.29–1.01
Interspecific competition (8)	0.47–1.46	0.73–2.10	0.48–1.35
Interspecific competition (12)	0.51–1.22	1.01–2.48	0.50–1.23

These shifts in isotopic positions resulted in some divergence in the isotopic niches of each species between allopatry and sympatry. In allopatry, *T. tinca* shared 39% of their isotopic niche with *C. auratus*, but this reduced to 14% in sympatry; *T. tinca* only shared 2% of their isotopic niche with *C. carpio* in allopatry, but this reduced to 0.3% in sympatry (Figure S4). In contrast, *C. carpio* shared 74% of their niche with *C. auratus*, and only reduced to 52% in sympatry (Figure S4). In the three interspecific competition treatments (Table 1), *T. tinca* no longer shared any of their isotopic niche with either non‐native species, whereas the extent of shared *C. carpio* niche with *C. auratus* was reduced to between 15% and 26% (Figure S5).

### 3.2 Trophic impacts of intra‐ vs. interspecific competition

**Figure 2:**
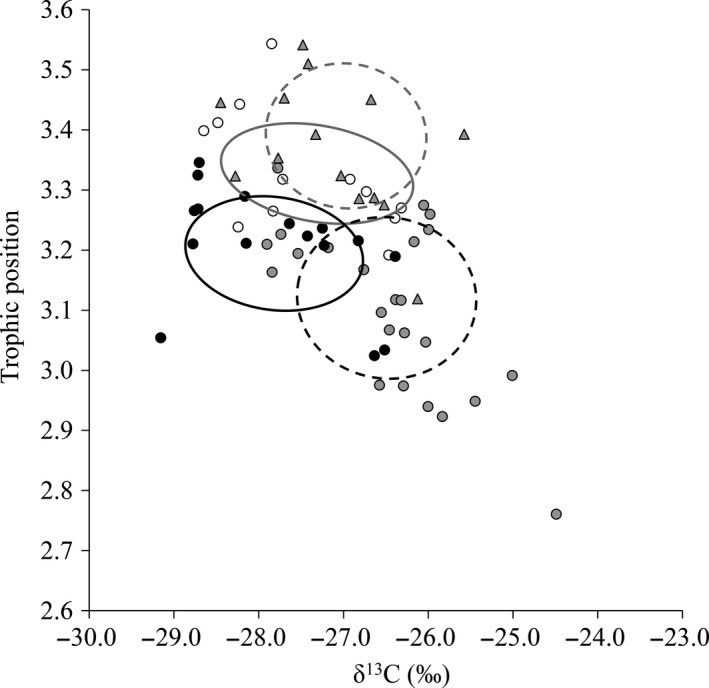
Comparison of the isotopic niche (as SEA_c_) of *Tinca tinca* in the experiment according to: Allopatry (black circles, solid black line), intraspecific competition (grey circles, dashed black line), interspecific competition (4) (clear circles, grey line) and interspecific competition (8) (grey triangles, grey dashed line) (*cf*. Table 1)

The LMEMs testing differences in *T. tinca* δ^13^C and TP between allopatry and the intra‐ and interspecific competition experimental treatments were significant (*p* < .01; Table S4). For δ^13^C, there was a significant difference between allopatry and the intraspecific competition treatment (allopatry: −27.72‰ ± 0.51‰; intraspecific competition: −26.25‰ ± 0.54‰; *p* < .01), and between allopatry and the interspecific competition (12) treatment (−25.82‰ ± 0.38‰, *p* < .01) (Table S4; Figure 1). Their TP was significantly higher in allopatry than the intraspecific competition treatment (allopatry: 3.21‰ ± 0.06‰; intraspecific competition: 3.10 ± 0.05; *p* = .05; Table S4; Figure 2). In the interspecific competition treatments, the *T. tinca* niche shift was to higher trophic positions compared to allopatry, with these differences significant in the (8) and (12) treatments (*p* ≤ .05; Table S4; Figure 2). In the treatments when numbers of fish were equal (*N* = 12), allopatric TP (3.10 ± 0.05) was significantly lower than when in competition with the two non‐native fishes (3.33 ± 0.07) (*p* < .01; Figure 2). Regarding overlaps in isotopic niches (as SEA_c_), the intraspecific competition treatment shared 15% of their niche with the allopatric treatment and 3% with the interspecific competition (4) treatment (Figure 2); this reduced to 0% for interspecific competition (12) treatment.

### 3.3 Impacts of intra‐ and interspecific competition on specific growth rates

**Figure 3:**
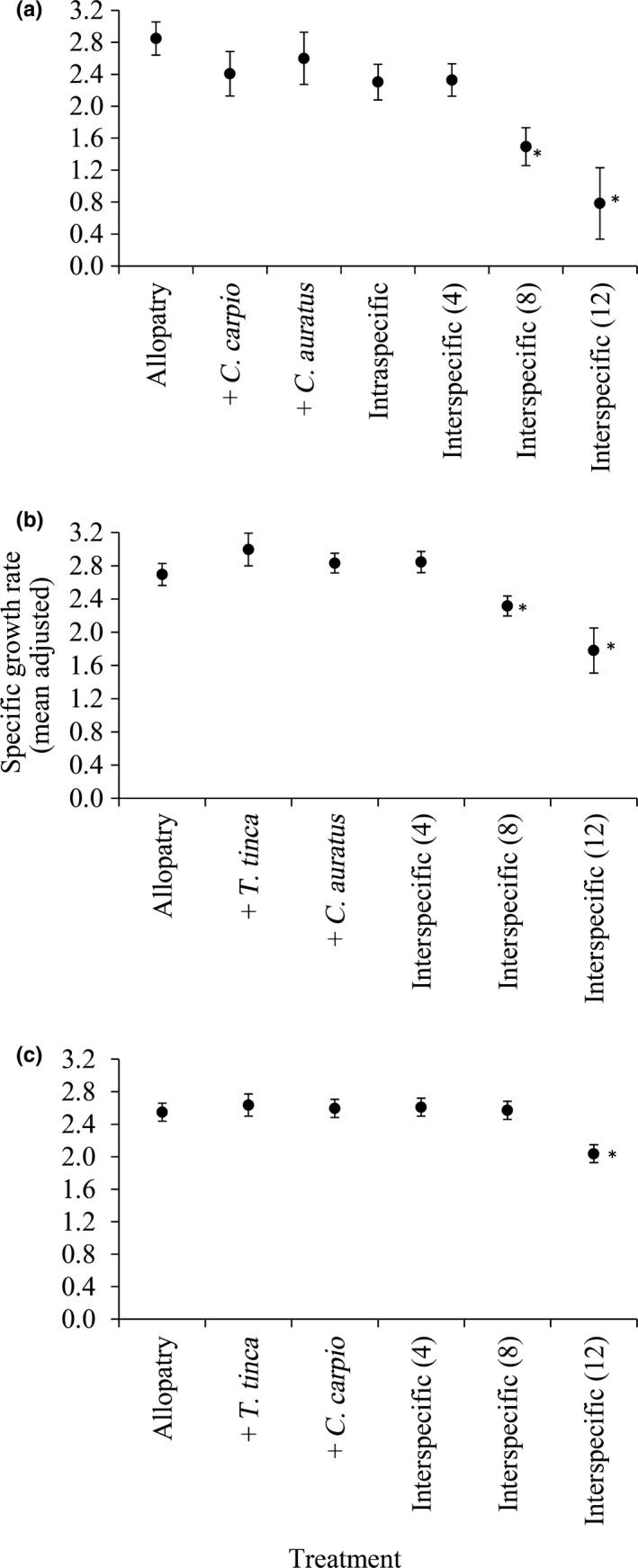
Mean specific growth rate by experimental treatment (adjusted for the effect of starting mass in *Tinca tinca* and *Cyprinus carpio* in the generalised linear models) for: (a) *Tinca tinca*, (b) *Cyprinus carpio* and (c) *Carassius auratus*. The treatments on the X axis are as per Table 1. *Difference between Allopatry and the treatment is significant at *p* < .01. Error bars represent 95% confidence limits

In each experimental treatment, there was an increase in total fish mass in each species across the 150 days and thus all mean SGRs were positive (Figure 3). The GLM testing the effect of treatment on SGR was significant for each species (*T. tinca*: Wald χ^2^ = 139.39, *df* = 6, *p* < .01; *C. carpio*: Wald χ^2^ = 35.50, *df* = 5, *p* < .01; *C. carassius*: Wald χ^2^ = 13.73, *df* = 5, *p* = .02). The effect of starting mass as a covariate on SGR was significant for *T. tinca* (*p* < .01) and *C. carpio* (*p* = .02) and so it was retained in their final models. It was not significant for *C. auratus* (*p* = .48) and so it was removed from their final model. Pairwise comparisons of differences in mean SGR between allopatry and the other treatments revealed that for each species, significantly decreased SGR was only apparent in the interspecific competition (8) and (12) treatments (*p* < .01) (Figure 3).

### 3.4 Trophic relationships in the invaded communities vs. the experiment

**Figure 4:**
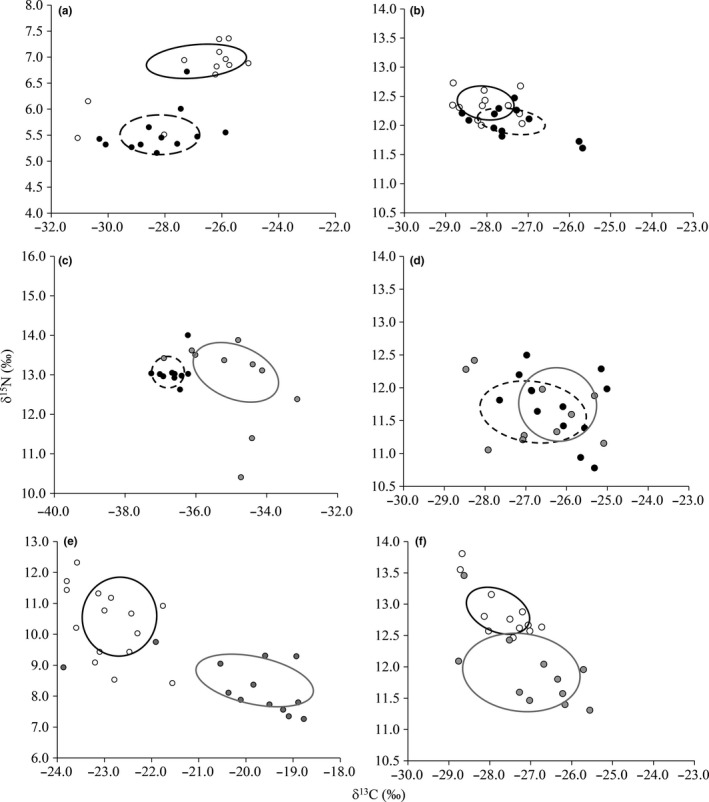
Stable isotope biplots comparing the isotopic niches (as SEA_c_) of sympatric *Tinca tinca*,* Cyprinus carpio* and *Carassius* spp. between the invaded communities and the experiment. Plots a, c and e are Field sites 1, 2 and 3 respectively. Plots b, d and f are the comparator sympatric treatments from the field experiment. *T. tinca*: clear circles and solid black line; *C. carpio*: grey circles, grey line; and *C. auratus*: black circles, black dashed line. Error bars represent 95% confidence limits. Note differences in the X and Y axes in a, c and e

In the invaded communities of Sites 1 and 3, there were no significant differences in the isotopic niche sizes of the sympatric model fishes (Site 1: *T. tinca*: 1.92 to 3.01‰^2^, *C. auratus*: 1.64 to 2.41‰^2^; Site 3: *T. tinca*: 3.04 to 4.27‰^2^, *C. carpio*: 2.97 to 4.03‰^2^) (Figure 4). In Site 2, however, the isotopic niche of *C. carpio* (3.12 to 4.70) was significantly higher than the *Carassius* spp. (0.94 to 1.89‰^2^) (Figure 4). In the invaded communities, the isotopic niches of fishes were highly divergent with no overlap. While they showed some consistency with the patterns identified within the experiment, their niches were also more divergent (Figure 4). There were also similarities in the relative positions of their niches in isotopic space between the experiment and invaded communities (Figure 4). For example, in both the invaded communities and the experiment, the isotopic niche of *T. tinca* was at a higher trophic position than *C. carpio*, but had similar values of δ^13^C (Figure 4). The sympatric treatment of *T. tinca* and *C. auratus* was the least similar to their pattern in the invaded communities, although the *T. tinca* niche was at the higher trophic position in both contexts (Figure 4).

## 4 DISCUSSION

The results of the experiment revealed that increased competition did not cause any significant shifts in isotopic niche sizes, contrary to Prediction 1. Instead, divergence in the isotopic niches of the fishes occurred, with this independent of shifts in somatic growth rates. Both increased intra‐ and interspecific competition impacted the isotopic niche of the model native species, as per Prediction 2. However, the impact on the isotopic niche differed between the competition types. Increased interspecific competition resulted in the niche shifting to a significantly higher trophic position, whereas increased intraspecific competition caused the niche to shift towards elevated δ^13^C. Finally, there were similar patterns of trophic niche divergence between the model species in the experiment and invaded communities, as per Prediction 3. This suggests that experimental predictions can help the understanding of how trophic relationships develop in invaded communities in the wild.

Ecological theory relating to invasions posits that invaders can out‐compete similar native competitors by occupying a broader niche (Elton, 1958). This has been supported empirically in studies involving non‐native taxa such as invasive crayfish (e.g. Ercoli, Ruokonen, Hämäläinen, & Jones, 2014; Olsson, Stenroth, Nyström, & Graneli, 2009). By occupying a broad niche, theory suggests that the invader suppresses the niche size of trophically analogous native species (Jackson, Grey, et al., 2016; Thomson, 2004). Conversely, other studies have suggested that when in sympatry, the trophic niche of both the invader and native species will constrict due to dietary specialisations (Jackson, Grey, et al., 2016; Tran et al., 2015a, 2015b). In the experiment, there were no significant shifts in the trophic niche sizes of each species between their treatments. This finding is, therefore, contrary to these aspects of invasion theory. Where an invader competes for the same resources as being used by a native species then, if that invader is a superior competitor, it has been predicted that their interactions will competitively exclude the native species. The can result in a shift in the position of the niche of the native species, potentially resulting in reduced food intake and suppressing growth rates (Bøhn et al., 2008). The experiment results had some consistency with this prediction, as all treatments resulted in a change in the position of the trophic niche of *T. tinca*. As invader abundance increased in the interspecific competition treatments, the extent of niche divergence also increased. While this suggests some competitive exclusion driven by *C. carpio* and *C. auratus*, some niche shifts were also apparent in the intraspecific competition treatment, suggesting the presence of some density‐dependent effects. However, the divergent niches developed independently of changes in fish growth rates, suggesting they enabled the fishes to maintain their food intake rates.

The results of the experiment were also consistent with patterns of interspecific trophic niche divergence detected in other invasive fishes. For example, Tran et al. (2015a, 2015b) revealed that in allopatry, the diet of the Asian invasive fish *Pseudorasbora parva* had the potential to overlap with some native fishes, but this never occurred in sympatry. Niche divergence was also apparent between non‐native pumpkinseed *Lepomis gibbosus* and native fishes in both rivers and ponds (Copp et al., 2017; Jackson, Britton, et al. 2016). Jackson and Britton (2014) detected partitioning between the trophic niches of sympatric *P. parva*,* C. carpio* and signal crayfish *Pacifastacus leniusculus* in ponds. In entirety, these results suggest that, in freshwater fishes at least, the initial response to an invasion is trophic niche divergence, leading to niche partitioning. This response reduces the strength of the competitive interactions, and can occur independently of shifts in niche size and growth rates. This response also occurs despite the high functional similarity of many of these fishes. Their traits must thus be sufficiently different or plastic between the species to enable these dietary specialisations to develop in sympatry (Jackson & Britton, 2014; Jackson, Britton, et al. 2016; Tran et al., 2015a, 2015b).

Recently, studies have suggested that ‘native invasions’, such as where wild populations are supplemented by hatchery‐reared conspecifics, can result in similar, and sometimes stronger, ecological impacts than those caused by non‐native invasions (Buoro et al., 2016; Carey et al., 2012). In the experiment, the comparison of intra‐ vs. interspecific competition across the treatments was also a simulation of a ‘native’ vs. ‘non‐native’ invasion, where the driver of impact was from increased competitive interactions. While increased intra‐ and interspecific competition both impacted the isotopic niche of *T. tinca*, there were differences in how these impacts manifested. Increased intraspecific competition caused the isotopic niche of *T. tinca* to shift to a significantly lower trophic position that was significantly carbon enriched. In contrast, increased interspecific competition resulted in the isotopic niche of *T. tinca* shifting to a significantly higher trophic position, with this also apparent in the invaded communities. While these results suggest that both ‘native’ and ‘non‐native’ invasions can indeed impact native species (*cf*. Buoro et al., 2016), they indicate that the impacts might differ between the invasion types (i.e. native vs. non‐native). This finding has potential implications for understanding how the model fishes can be better used in fishery enhancement schemes, particularly regarding the numbers being introduced in relation to the ecological effects they might incur (Bašić & Britton, 2016). The experiment could not, however, determine how these differences between intra‐ and interspecific competitive differences developed temporally. It is therefore recommended that this is explored in future work, such as through more controlled experiments using a wider range of model species (e.g. Dick et al., 2017).

Each natural fish community had been invaded by either *C. carpio* or *Carassius* spp. Within these multispecies communities, each sympatric combination of the model species had isotopic niches that were divergent, with no sharing of isotopic space between them. This might have been the result of differences in the length ranges of the fishes in each site, especially in Site 3, resulting in the different size classes of fish exploiting different food resources. However, these patterns were relatively consistent with those detected in the experiment. They were also consistent with other studies on invasive fishes that suggest isotopic niche partitioning, rather than niche convergence, is the general pattern within invaded fish communities (e.g. Bašić & Britton, 2016; Jackson & Britton, 2014; Tran et al., 2015a, 2015b), except perhaps where the invader has attained high population abundances (e.g. Britton, Davies, & Harrod, 2010). The data from the invaded communities have the caveat that they were non‐replicated wild samples that were only sampled once, and were subject to uncontrolled environmental conditions. Comparison of the results between the experiment and the invaded communities did, however, indicate that when the model fishes were in the wild they exhibited complete partitioning in their isotopic niches, a contrast to the experiment. This might relate to the experiment being completed in relatively enclosed spaces, resulting in reduced opportunities for exploiting different food resources. The invaded communities were also more complex with higher species richness and so might have contributed to their niche partitioning through stronger interspecific competition. Also, the experimental data were collated in relatively controlled conditions and over shorter timeframes than the invaded communities. Indeed, ecological experiments often have ‘scaling‐up’ issues that arise from their limited timeframes (Korsu, Huusko, & Muotka, 2009; Spivak, Vanni, & Mette, 2011). Mesocosm approaches have, however, been used successfully for understanding the trophic relationships of freshwater fishes (e.g. Bašić & Britton, 2016; Jackson, Pegg, Allen, & Britton, 2013), with these successfully extrapolated to wild populations to help explain ecological patterns (e.g. Copp et al., 2017; Tran et al., 2015a, 2015b).

In summary, the experiment revealed isotopic niche divergence developed between the model fishes when intra‐ and interspecific competition was elevated. The magnitude and direction of niche divergence in the model native fish did, however, differ between intra‐ and interspecific competition. Patterns of trophic niche partitioning were also strongly apparent between the model fishes in the invaded communities. As isotopic niche divergence occurred in the experiment in isolation from niche constriction and impacts on growth rates, this suggests the initial ecological response to an introduction is the trophic re‐organisation of the food web to minimise the interactions between the competing species.

## AUTHORS’ CONTRIBUTIONS

J.R.B. and A.R.‐N. conceived the ideas and designed methodology; J.R.B. and H.V. collected the data; J.R.B. and F.A.‐T. analysed the data; J.R.B. and F.A.‐T. led the writing of the manuscript. All authors contributed critically to the manuscript and gave final approval for publication.

## DATA ACCESSIBILITY

Data available from the Dryad Digital Repository: Experiment: https://doi.org/10.5061/dryad.qn186 (Britton, Ruiz‐Navarro, Verreycken, & Amat, 2017); Invaded communities: https://doi.org/10.5061/dryad.12344 (Tran et al., 2015b).
