# Supplementary material for: Trophic consequences of introduced species: Comparative impacts of increased interspecific versus intraspecific competitive interactions
Source: Funct Ecol. 2017 Sep 21;32(2):486–95. doi: 10.1111/1365-2435.12978 (PMC5856055; doi:10.1111/1365-2435.12978)
Supplement: Supplementary file 1 [file FEC-32-486-s001.pdf]

Functional Ecology

BRITISH ECOLOGICAL SOCIETY

Trophic consequences of introduced species

Robert Britton, Ana Ruiz-Navarro, Hugo Verreycken and Fatima Amat-Trigo

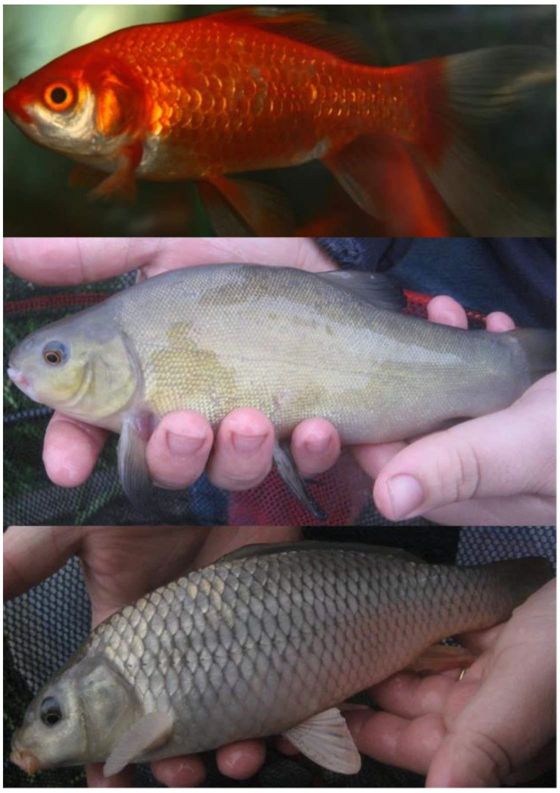

Photo provided by authors.

Species are regularly moved from regions where they are found naturally into new regions. These 'non-native' species can then cause considerable ecological disruption ('impact'). For example, when non-native and native species share the same food items then they compete ('inter-specific' competition). This can impact both species, as they might no longer be able access the food required to maintain their energy requirements. Consequently, the species might change the food items they consume - their 'trophic niche' - to reduce competition. These niches might increase in size (greater range of food items) or decrease (smaller range of food items). Alternatively, they might remain of similar size but comprise of alternative food items ('niche divergence').

Whilst there has been concern about the impacts of non-native species, there has been a recent focus on how releases of native species can also cause ecological impacts. These releases are often intended to increase population sizes, such releasing fish into freshwaters used for angling. Releasing numbers of the same species means there is potential for increasing competition for food within the population - 'intra-specific' competition. Studies have suggested the impacts of intra- and inter-specific competition could be similar.

Our study used an experiment to investigate this, using a native freshwater fish and two non-native fishes. We demonstrated that increasing both competition types did not affect the sizes of the fish trophic niches. However, we revealed that as both competition types increased, they impacted the trophic niches of all species by altering the food items the fish consumed (niche divergence). The manner in which this occurred differed between intra- and inter-specific competition. For example, in the native fish, increased intra-specific competition shifted their diet towards being more plant based, whereas inter-specific competition caused this to be more invertebrate based. These changes also occurred without the growth rates of the fishes changing, suggesting they maintained their food intake levels. We then demonstrated that these 'trophic' patterns in our experiment results were also apparent in wild populations of the native and non-native fishes, thus helping to explain the ecological relationships of native and non-native species in the wild.
