# Supplementary material for: Trophic consequences of introduced species: Comparative impacts of increased interspecific versus intraspecific competitive interactions
Source: Funct Ecol. 2017 Sep 21;32(2):486–95. doi: 10.1111/1365-2435.12978 (PMC5856055; doi:10.1111/1365-2435.12978)
Supplement: Supplementary file 2 [file FEC-32-486-s002.docx]

**Appendix S1: Supplementary tables and figures**

Table S1. Outputs of the linear mixed model testing the differences in δ^13^C and trophic position (TP) of *Tinca tinca* between the treatments of the experiment, where replicate number was the random effect on the intercept. Errors around the mean are 95% confidence limits.

δ^13^C

| Overall model result: AIC = 199.76; log likelihood = 195.76; P < 0.01. | | |
| --- | --- | --- |
| Pairwise comparison | | Mean difference  (estimated marginal means) |
| Allopatric *T. tinca* | *T. tinca* sympatric with *C*. *carpio* | 0.17 ± 0.95, P = 1.00 |
|  | *T. tinca* sympatric with *C. auratus* | 0.15 ± 1.08, P = 1.00 |
|  | *T. tinca* Inter-specific competition (4) | 0.27 ± 1.08, P = 1.00 |
|  | *T. tinca* Inter-specific competition (8) | 0.69 ± 0.91, P = 0.37 |
|  | *T. tinca* Inter-specific competition (12) | 1.93 ± 0.82, P < 0.01 |

TP

| Overall model result: AIC = -109.91; log likelihood = -113.91; P < 0.01. | | |
| --- | --- | --- |
| Pairwise comparison | | Mean difference  (estimated marginal means) |
| Allopatric *T. tinca* | *T. tinca* sympatric with *C*. *carpio* | 0.14 ± 0.12, P = 0.01 |
|  | *T. tinca* sympatric with *C. auratus* | 0.04 ± 0.16, P = 1.00 |
|  | *T. tinca* Inter-specific competition (4) | 0.10 ± 0.14, P = 0.40 |
|  | *T. tinca* Inter-specific competition (8) | 0.20 ± 0.12, P < 0.01 |
|  | *T. tinca* Inter-specific competition (12) | 0.08 ± 0.10, P = 0.70 |

Table S2. Outputs of the linear mixed model testing the differences in δ^13^C and trophic position (TP) of *Cyprinus carpio* between the treatments of the experiment, where replicate number was the random effect on the intercept. Errors around the mean are 95% confidence limits.

δ^13^C

| Overall model result: AIC = 262.20; log likelihood = 258.20; P < 0.01. | | |
| --- | --- | --- |
| Pairwise comparison | | Mean difference  (estimated marginal means) |
| Allopatric *C*. *carpio* | *C*. *carpio* sympatric with *T. tinca* | 0.60 ± 1.33, P = 1.00 |
|  | *C*. *carpio* sympatric with *C. auratus* | 0.24 ± 1.36, P = 1.00 |
|  | *C*. *carpio* Inter-specific competition (4) | 0.26 ± 1.50, P = 1.00 |
|  | *C*. *carpio* Inter-specific competition (8) | 0.97 ± 1.23, P = 0.29 |
|  | *C*. *carpio* Inter-specific competition (12) | 1.74 ± 1.15, P < 0.01 |

TP

| Overall model result: AIC = -65.15; log likelihood = -69.15; P = 0.47. | | |
| --- | --- | --- |
| Pairwise comparison | | Mean difference  (estimated marginal means) |
| Allopatric *T. tinca* | *C*. *carpio* sympatric with *T. tinca* | 0.01 ± 0.16, P = 1.00 |
|  | *C*. *carpio* sympatric with *C. auratus* | 0.08 ± 0.06, P = 1.00 |
|  | *C*. *carpio* Inter-specific competition (4) | 0.01 ± 0.06, P = 1.00 |
|  | *C*. *carpio* Inter-specific competition (8) | 0.07 ± 0.15, P = 1.00 |
|  | *C*. *carpio* Inter-specific competition (12) | 0.05 ± 0.14, P = 1.00 |

Table S3. Outputs of the linear mixed model testing the differences in δ^13^C and trophic position (TP) of *Carassius auratus* between the treatments of the experiment, where replicate number was the random effect on the intercept. Errors around the mean are 95% confidence limits.

δ^13^C

| Overall model result: AIC = 245.87; log likelihood =241.87; P < 0.01. | | |
| --- | --- | --- |
| Pairwise comparison | | Mean difference  (estimated marginal means) |
| Allopatric *C*. *auratus* | *C*. *auratus* sympatric with *T. tinca* | 0.22 ± 1.22, P = 1.00 |
|  | *C*. *auratus* sympatric with *C. carpio* | 1.01 ± 1.06, P = 0.07 |
|  | *C*. *auratus* Inter-specific competition (4) | 0.12 ± 1.22, P = 1.00 |
|  | *C*. *auratus* Inter-specific competition (8) | 1.18 ± 0.97, P = 1.00 |
|  | *C*. *auratus* Inter-specific competition (12) | 1.32 ± 0.90, P < 0.01 |

TP

| Overall model result: AIC = -116.36; log likelihood = -120.36; P < 0.01. | | |
| --- | --- | --- |
| Pairwise comparison | | Mean difference  (estimated marginal means) |
| Allopatric *C*. *auratus* | *C*. *auratus* sympatric with *T. tinca* | 0.02 ± 0.13, P = 1.00 |
|  | *C*. *auratus* sympatric with *C. carpio* | 0.11 ± 0.11, P = 0.05 |
|  | *C*. *auratus* Inter-specific competition (4) | 0.11 ± 0.15, P = 0.51 |
|  | *C*. *auratus* Inter-specific competition (8) | 0.03 ± 0.10, P = 1.00 |
|  | *C*. *auratus* Inter-specific competition (12) | 0.18 ± 0.13, P < 0.01 |

Table S4. Outputs of the linear mixed model testing the differences in δ^13^C and trophic position (TP) of *Tinca tinca* between allopatry and the intra-specific and inter-specific competition treatments of the experiment, where replicate number was the random effect on the intercept. Errors around the mean are 95% confidence limits.

δ^13^C

| Overall model result: AIC = 219.47; log likelihood = 215.47; P < 0.01. | | |
| --- | --- | --- |
| Pairwise comparison | | Mean difference  (estimated marginal means) |
| Allopatric *T. tinca* | Intra-specific competition | 1.42 ± 0.81, P < 0.01 |
|  | Inter-specific competition (4) | 0.21 ± 1.09, P = 1.00 |
|  | Inter-specific competition (8) | 0.77 ± 0.94, P = 1.00 |
|  | Inter-specific competition (12) | 1.86 ± 0.84, P < 0.01 |

TP

| Overall model result: AIC = -100.17; log likelihood = -104.17; P < 0.01). | | |
| --- | --- | --- |
| Pairwise comparison | | Mean difference  (estimated marginal means) |
| Allopatric *T. tinca* | Intra-specific competition | 0.11 ± 0.11, P = 0.06 |
|  | Inter-specific competition (4) | 0.13 ± 0.14, P = 0.10 |
|  | Inter-specific competition (8) | 0.16 ± 0.12, P < 0.01 |
|  | Inter-specific competition (12) | 0.11 ± 0.11, P = 0.04 |

Figure S1. Distribution of the individual stable isotope data points of *Tinca tinca* in each replicate per experimental treatment (black circle: Replicate 1; clear circle: Replicate 2; grey circle: Replicate 3).

Figure S2. Distribution of the individual stable isotope data points of *Cyprinus carpio* in each replicate per experimental treatment (black circle: Replicate 1; clear circle: Replicate 2; grey circle: Replicate 3).

Figure S3. Distribution of the individual stable isotope data points of *Carassius auratus* in each replicate per experimental treatment (black circle: Replicate 1; clear circle: Replicate 2; grey circle: Replicate 3).

Figure S4. Comparison of the isotopic niche (as SEA_c_) (main plots) and extent of the niche overlap (inset text, %) of the model species in the experiment between Allopatry (n = 8) and their Sympatry treatments (n = 4+4) (*cf*. Table 1). *Tinca tinca* vs. *Cyprinus carpio*: A = allopatry, B = sympatry. *T. tinca* vs. *Carassius auratus*: C = allopatry, D = sympatry. *C. carpio* vs. *C. auratus*: E = allopatry, F = sympatry. *T. tinca*: clear circles and solid black line; *C. carpio*: grey circles, grey line; and *C. auratus*: black circles, black dashed line.

Figure S5. Comparison of the isotopic niche (as SEA_c_) (main plots) and extent of the overlap (inset text, %) of the model species between their Allopatry experimental treatments (A), and in the three inter-specific competition treatments, where B: inter-specific competition (4), C: inter-specific competition (8), and D: inter-specific competition (12) (*cf*. Table 1). *T. tinca*: clear circles and solid black line; *C. carpio*: grey circles, grey line; and *C. auratus*: black circles, black dashed line.
